# Quality of Cancer Care in Switzerland: Going Beyond Traditional Quality Indicators by Collecting Patient-Reported Experiences of Cancer Care

**DOI:** 10.3389/phrs.2022.1604813

**Published:** 2022-05-17

**Authors:** Chantal Arditi, Isabelle Peytremann-Bridevaux

**Affiliations:** Center for Primary Care and Public Health (Unisanté), Department of Epidemiology and Health Systems, University of Lausanne, Lausanne, Switzerland

**Keywords:** patient-reported measures, experiences of care, cancer care, quality of care, patient surveys, patient satisfaction, Switzerland

## Abstract

**Background:** High-quality cancer care should be effective, safe, accessible, efficient, equitable, and responsive to patients’ needs. In Switzerland, information on the safety and effectiveness of cancer care is available, but not on responsiveness. Systematic and comprehensive reports from patients on cancer care are missing and needed to complete the assessment of the quality of cancer care.

**Evidence:** Patient-reported experiences of cancer care are key to evaluate responsiveness of care and drive quality improvement initiatives in oncology practice. Studies have found that responsive care leads to more positive experiences of care, which can lead to more effective treatments and health benefits.

**Policy Options and Recommendations:** Our first recommendation is to develop a position statement on the importance and value of patient-reported experiences of cancer care. Our second recommendation is to systematically collect patients’ experiences of cancer care at the national level, through a dedicated national cancer-specific measurement program or through the integration of patient-reported experiences measures in cancer registries.

**Conclusion:** The systematic collection of patient-reported experiences of cancer care provides essential information on what matters to patients in addition to traditional clinical information, including patients as partners of the overall assessment of healthcare performance.

## Background

One of the main objectives of a healthcare system is to improve the care and experience of care of people going through the system [1], by providing high-quality responsive care (also called patient- or people-centered care). Such care is defined as care delivered in a way that responds to patients’ physical, emotional, social and cultural needs, where interactions with health professionals are compassionate and empowering, and where patients’ values and preferences are taken into account [2, 3]. Responsive care is especially important in cancer care, as cancer has a particular emotional, social and financial burden on patients and their families, in addition to the health burden. Responsiveness of care is also one of the six core dimensions of quality of care according to the widely used framework developed by the Organisation for Economic Co-operation and Development (OECD), along with effectiveness, safety, accessibility, efficiency and equity [4, 5].

In Switzerland, most efforts have focused on the collection of quality indicators pertaining to the effectiveness and safety dimensions of cancer care within acute care hospitals, such as annual quality indicators for acute care hospitals (e.g., number of patients treated for colorectal cancer) and mortality rates (e.g., mortality rates for patients with breast cancer who had had breast resection surgery). Systematic and comprehensive reports from cancer patients themselves are missing. This is unfortunate since these reports are needed to complete the assessment of the quality of cancer care and evaluate whether current cancer care responds to the needs of patients across the care continuum, from the screening process to remission and follow-up.

### Definition of Patient-Reported Experience Measures

Patients can report not only on their health—whether the treatment reduced their pain, for example, or if it helped them live more independently—but also on their experience of being treated—whether the treatment was properly explained, for example, or if they felt involved in decisions about their care. The umbrella term “patient-reported measures” refers to both types of reports, that come directly from the patient without interpretation by a physician or anyone else [6] and are usually collected with standardized surveys. While patient-reported outcome measures (PROMs) assess the health result of care received (e.g., symptoms, quality of life), patient-reported experience measures (PREMs) assess experiences with the delivery of care (e.g., communication with nurses and doctors, discharge coordination—see Table 1) [7].

**TABLE 1:** Definition of patient-reported outcome measures (PROMs) and patient-reported experience measures (PREMs) (Switzerland, 2021).

PROM: a measure of patients’ perception of their health, symptoms, functioning, well-being and quality of life, to evaluate the impact of care on health and well-being according to patients	PREM: a measure of patients’ perception of their experience of care focusing on the delivery of care, to evaluate the quality and patient-centeredness of care according to patients
Generic PROMs are intended to make comparisons between and within interventions, and across different diseases and sectors of care. They often focus on the person’s health state, on “health-related quality of life (HRQoL)” or “Quality of Life (QoL)” in general	PREMs encompass the range of interactions that patients have with the health system relating to their satisfaction (e.g., with information given by nurses and doctors); subjective experiences (e.g., staff helped with pain); objective experiences (e.g., waiting time before appointment); and observations of healthcare providers’ behavior (e.g., whether or not a patient was given discharge information)
Condition-specific PROMs are specific to a particular disease (e.g., diabetes), a domain (e.g., pain), or an intervention (e.g., knee arthroplasty). They are more sensitive to small, yet clinically significant, changes in specific patient populations than generic PROMs, but they do not allow comparisons across diseases or populations

### Purposes and Uses of Patient-Reported Experience Measures

PREMs have been increasingly collected worldwide, in clinical, economic and health services research, as well as in general assessments of health services and system performance. They have different purposes and uses at different levels. At the patient (micro) level, real-time patient feedback on experiences of care are usually collected at point-of-care, providing clinicians and other healthcare professionals with the opportunity to address concerns and improve perceptions and processes of care immediately [8]. At the institutional (meso) level, aggregated PREMs are used to drive quality improvement initiatives. They are also used to compare the performance of providers (benchmarking), to identify which care aspects insufficiently addressed, and to inform the general public to enable informed patient choice (public reporting) [9, 10]. At the national (macro) level, PREMs are used for monitoring responsiveness of health services, for reimbursement decisions and payments models, and for macro-level healthcare performance measurements, for example. The value of population level measures increases when they are linked to other surveillance data, such as clinical registries, billing and hospital discharge data.

### Methods to Collect Patient-Reported Experience Measures

There are quantitative and qualitative methods to measure and collect patients’ experience of care. Surveys using structured self-completed questionnaires, given or sent to patients at a single or multiple points in time, are the most common form of quantitative measures of patients’ experience. These surveys are designed to produce numerical data that can be analyzed statistically. Their emphasis is on examining patterns and trends from a large sample, providing large coverage and ability to compare, but often lacking depth because questions and response options are predetermined [9]. An important limitation of surveys is also that some patient groups are consistently underrepresented: those who do not speak the national language or with low (health) literacy and those with poor prognosis [11]. In addition, the way patients evaluate their experiences can be influenced by their socio-demographic characteristics (age, sex, income level), expectations, preferences, personality, previous experiences, as well as their health status, for instance [12]. Consequently, careful evaluation of risk adjustment strategies is required when patient experiences are compared across populations and providers.

Patients’ experiences of care can also be collected through qualitative reports, such as patient stories, complaints and compliments, focus groups or interviews. The focus of these qualitative methods is on obtaining an in-depth understanding of people’s experiences and the way they explain or interpret these [9]. However, these qualitative methods also have limitations: they require more time and expertise to collect and analyze data; they are more likely to reflect individual issues rather than more general and systematic issues; comparisons across populations and providers (e.g., hospitals) and over time are difficult to make; and results are less generalizable than quantitative results.

### Reporting of Patient-Reported Experience Measures

Reporting can include instant alerts to healthcare professionals when using real-time feedback but also public reporting on website to inform consumers and publication of these measures in quality reports. The public reporting of patients’ experiences of care is important, as it is seen as an important mechanism for “holding providers to account for the quality of care (“voice”) and for empowering patients to act as discerning consumers (“choice”)” [7].

## Evidence

We searched the scientific literature for systematic reviews on the validity and reliability of PREMs, the effectiveness of their use to improve the quality of care and the association between positive experiences of care and patient outcomes.

Patient-reported experience measures need to be valid and reliable to be used for quality assessment of healthcare services, in combination with other aspects, such as the clinical relevance of the instrument and the domains of patient-reported experience that the instrument covers. In a recent systematic review of 88 instruments measuring patient experiences in healthcare in general [13], the authors reported that seven of the 10 validity and reliability criteria were not undertaken in more than half of the instruments. Also, information on responsiveness, an instrument’s ability to detect changes overtime, was lacking for over 90% of them.

Two systematic reviews exploring how PREMs were collected, communicated and used to inform quality improvement initiatives [14, 15] concluded there was limited evidence on the effectiveness of interventions informed by patient feedback, as few studies were well-designed trials. In addition, one of these reviews showed that there was no single best way to collect or use patient experience data for quality improvement [14].

Additionally, we identified three reviews that investigated the association between patient experiences of care and patient outcomes. The first review concluded that patient experiences were positively associated with clinical effectiveness and patient safety, and supported the case for the inclusion of patient experiences as one of the central pillars of quality in healthcare [16]. The second review concluded that better patient care experiences were associated with higher levels of adherence to recommended prevention and treatment processes, better clinical outcomes, better patient safety within hospitals, and less healthcare utilization [17]. In the third review looking at the link between patient experiences and cancer survival, patients’ satisfaction, psychosocial support, and satisfaction with quality of life were associated with survival. However, authors cautioned about the methodological complexity of determining the relationship between cancer patient experience and subsequent survival [18].

## Policy Options and Recommendations

Based on the literature presented above, this policy brief includes two recommendations to going beyond traditional quality indicators by collecting patient-reported experiences of cancer care. These recommendations were also discussed during a stakeholder dialogue that took place in November 2020 with eleven stakeholders representing patient associations, professional associations, educational institutions, quality associations, and hospitals.

The first recommendation is to develop and publish a position statement on the importance and value of patient-reported experiences of cancer care. This is useful to provide guidance for future initiatives on this topic and promote similar developments for other chronic conditions. Stakeholders agreed with this recommendation and added that it will push forward the importance of patients’ experiences of care in the political agenda, clarify the concept of patients’ experiences of care, and shed light on various stakeholders’ interests. They suggested that the intended audience, objective of the statement and leadership would need careful preparation.

The second recommendation is to systematic collect, analyze and report patients’ experiences of cancer care at a national level to gather the necessary data to evaluate responsiveness of cancer care and inform quality improvement policy and practice. We present two options for this recommendation, based on two frequent strategies emerging from the literature: the first strategy is to collect data using postal or online questionnaires among a sample of patients; the second strategy is to integrate patient-reported data in clinical registries, although this has so far mainly be done for outcomes of care reported by patients (PROMs) rather than experiences of care (PREMs).

### Option 1: National Cancer-Specific Measurement Program

The first option is to develop and implement a dedicated national cancer-specific measurement program collecting experiences of care. For Switzerland, two options for the instrument were identified: the Swiss cancer-specific survey from the Swiss Cancer Patient Experience (SCAPE) survey or the future OECD’s patient-reported indicators survey (PaRIS). The Swiss cancer-specific survey covering patient experiences across the care continuum was developed in 2018 for the multicenter SCAPE study in French-speaking Switzerland (www.scape-enquete.ch) [19] and later translated in German for the follow-up study SCAPE-2. The OECD’s PaRIS survey includes a PREM section covering generic and common aspects of people-centered care: accessibility, communication, shared decision-making, and continuity and coordination, as well as measures of health literacy and patient engagement and activation [20]. The stakeholders added during the dialogue that the choice of instrument depends on the potential aims of data collection. While the Swiss cancer-specific survey could be more impactful to influence clinical care through improvement initiatives, the international survey could allow international comparisons of overall care. Both instruments could be used in parallel, or combined, by developing indicators in the Swiss survey complementing those from the international survey.

The strategy of having a dedicated national measurement program on experiences of cancer care has been adopted in several countries. For instance, the annual National Cancer Patient Experience Survey (NCPES) launched by the National Health Service in England in 2010, was designed to monitor national progress on cancer care, to drive local quality improvements, to assist commissioners and providers of cancer care and to inform the work of the various charities and cancer stakeholders groups. Another example is the Consumer Assessment of Healthcare Providers and Systems (CAHPS) Cancer Care Survey in the USA, developed between 2009 and 2016 [21], which main purpose is to support the efforts of cancer centers and oncology practices to improve the patient-centeredness of cancer care, as well as to inform decisions made by providers and patients, for instance.

### Option 2: Integration of PREMs in Cancer Registries

The second option is to integrate the collection of PREMs within the Swiss cancer registries, which systematically collect clinical data on the type and stage of the disease and the first treatment since 2020. There was disagreement between the stakeholders around the relative importance and benefits of integrating PREMs versus PROMs. While some argued that PROMs would make more sense and would add more benefit, others argued that both were useful and fulfilling different objectives. Stakeholders discussed several areas of uncertainty, such as difficulties in merging datasets, high workload for collecting data, issues of pseudo-anonymization, legal obligations and data protection.

The strategy of collecting patient experiences through registries has been chosen in a few countries, such as Sweden which has set up over a 100 national quality registries, 40% of which collect a patient experience measure [22, 23]. Whereas the purpose is to develop and ensure the quality of care, these registries are also used for clinical research and public quality reporting. Another example comes from The Monash Partners Comprehensive Cancer Consortium (MPCCC) in Australia, currently piloting the collection of PROMs and PREMs from pancreatic cancer patients integrated within the Upper Gastrointestinal Cancer Registry [24].

### Implementation Considerations

The implementation of a national PREMs program or the integration of PREMs in registries should follow published guidelines and principles, such as those from the OECD [25]. The health department of New South Wales in Australia has also defined guiding principles within which patient-reported measures should operate [26]. Regarding registries, the 2020 updated AHRQ publication, “Registries for Evaluating Patient Outcomes: A User’s Guide” is a reference handbook with practical information on the design, operation, and analysis of patient registries and inclusion of patient-reported outcomes; it could be adapted to patient-reported experiences of care [27].

There are many barriers and facilitators reported in the literature for the implementation and use of patient’s experiences of care at the patient (micro), institutional (meso) and national (macro) levels, summarized in Table 2 [14, 28–33]. A recent Belgian report [29] highlighted the most important facilitators for successful PREMs implementation: a patient-centered healthcare culture supported by management and politics, awareness of the potential value of PREMs from the providers, involvement of patients in all steps, and sufficient resources. Availability and cost of human resources to collect PREMs data are also an important consideration for the implementation of PREMs, as well as consideration of privacy and ethical concerns. Moreover, an adequate IT infrastructure is needed to manage all the data, as well as the availability of people for the management and analysis of the data. The stakeholders additionally identified the following important facilitators: having simple, disease-specific and meaningful questions, using a short questionnaire tailored to patients’ literacy level, having electronic health solutions available, and having a clear objective of using results to implement change. In Switzerland, implementing a wide-scale and coordinated measurement of patient-reported experiences of cancer care would be particularly challenging because of three additional country-specific factors: Swiss federalism with the 26 cantons and 26 slightly different healthcare systems, the fragmented, complex, and mixed-financed healthcare system, as well as the need to consider three main national languages.

**TABLE 2:** Barriers and facilitators for the implementation of patient-reported experience measures (PREMs) (Switzerland, 2021).

Barriers	Facilitators
Patient (micro) level	
Questionnaire (Q) related	Questionnaire (Q) related
Length and complexity of Q	Parsimonious Q
Lack of availability of translated and culturally meaningful versions	Disease-specific and meaningful questions
Questions not relevant to patients’ issues	Simple questions and scales (e.g., scale with verbal descriptors)
Compliance issues in completing the Q	Translations available
Literacy issues	Involving patients in designing the Q
Technology (electronic questionnaire)	Technology (electronic questionnaire)
Comfort level with technology & the internet (if electronic)	IT support available
Technical problems during completion	
Concerns over confidentiality and security	
Privacy concerns	
Over confidentiality of answers	
Over potential identification	
Patient health condition & abilities	
Too ill to answer (response bias)	
Disability (e.g. sight, hands)	
Provider and institutional (meso) level	
Data collection and use	Data collection and use
Lack of understanding the interpretation of the aggregated results	High response rate
Poor specificity of results	Repeated measures over time
Poor perceived reliability and validity of the measure	Providing training on use and interpretation of aggregated PREMs
Administrative burden	Disseminating positive survey findings to boost morale
Response and selection bias	Organization and logistics
Organization and logistics	Working culture supportive of improvement, change and patient views
Not enough staff	Dedicated meeting time to present results
For electronic surveys: lack of patient emails	Patient-centered work culture
No integration of electronic results into electronic health records	Leadership by senior member or having a coordinator in charge
Providers’ beliefs & attitudes	Involving providers in the implementation process
Fear of change	Fully integrated electronic data
Feeling of being assessed and criticized in aggregated results	Communication
Lack of understanding of the added value of aggregated results	Providing timely feedback
Fear of increased workload	Providing results in an easily accessible format
Communication	Aggregated measures relevant to clinical management
Long delay between PREMs measurement and reporting	Financial
Technical problems when communicating the results	Financial incentives
Financial	
Not enough financial resources to implement program	
High cost of collecting PREMs by paper mailings	
Lack of time and knowledge to ensure scientific validation of the Q or financial means to outsources the scientific validation	
National health system (macro) level	
Tension among stakeholders regarding data use for different purposes	Adopting a common standard and metric
Conflicting or competing priorities (nationally, regionally, within organizations)	Acceptability of usefulness of measures
Lack of national conceptual framework including PREMs	Including the results in the performance management system and financial targets
Lack of risk- and case-mix-adjustment strategies	Central coordination
Lack of effective reporting strategies	Gradual implementation
Lack of interoperability between systems	Support from e-health
Complexity of integrated data collection	Legal basis
Privacy legislation	
Costs of developing a national program, providing training, implementing program, analyzing data, communicating data	

## Conclusion

In this policy brief, we proposed two recommendations to promote the collection and use of patient-reported experiences of care and present two options to collect actionable measures on cancer patients’ experiences of going through the healthcare system. Reports from patients on their experiences of care are essential to evaluate responsiveness of care, on the six key dimensions of quality of care. Indeed, the systematic collection of patient-reported experiences of cancer care enables to consider what matters to patients in addition to traditional quality indicators. It also includes patients as partners of the overall assessment of healthcare performance. However, only a few countries systematically collect this information. We present recommendations and options for Switzerland, but they are relevant for other countries as well. We focused on cancer care, as cancer is among the five most frequent non-communicable diseases in Switzerland and affects most individuals during their life course, either as a patient or as a caregiver to a family member or friend. However, the options presented for cancer care could be transferred and adapted to other frequent chronic conditions, such as diabetes and cardiovascular disease.

## Full Policy Brief

The full policy brief is available on the website of the Swiss Learning Health System (https://www.slhs.ch/policy-briefs-stakeholder-dialogues/our-topics/prems-in-cancer-care/), along with the summary of the stakeholder dialogue that took place in November 2020, when eleven stakeholders representing patient associations, professional associations, educational institutions, quality associations, and hospitals, discussed the content and recommendations of the policy brief.
